# Association between abnormal plasma metabolism and brain atrophy in alcohol-dependent patients

**DOI:** 10.3389/fnmol.2022.999938

**Published:** 2022-12-13

**Authors:** Zheyu Zhang, Sifang Zhang, Jianhua Huang, Xiaoyun Cao, Chao Hou, Zhihong Luo, Xiaoyan Wang, Xuejun Liu, Qiang Li, Xi Zhang, Yujun Guo, Huiqiong Xiao, Ting Xie, Xuhui Zhou

**Affiliations:** ^1^Department of Addiction Medicine, Hunan Institute of Mental Health, Brain Hospital of Hunan Province (The Second People’s Hospital of Hunan Province), Changsha, China; ^2^Department of Integrated Traditional Chinese & Western Medicine, The Second Xiangya Hospital, Central South University, Changsha, China; ^3^Hunan Academy of Chinese Medicine, Hunan University of Chinese Medicine, Changsha, China; ^4^The School of Clinical Medicine, Hunan University of Chinese Medicine, Changsha, China

**Keywords:** alcohol, brain atrophy, cognitive dysfunction, glycerophospholipid metabolism, metabolomic

## Abstract

**Objective:**

In this study, we aimed to characterize the plasma metabolic profiles of brain atrophy and alcohol dependence (s) and to identify the underlying pathogenesis of brain atrophy related to alcohol dependence.

**Methods:**

We acquired the plasma samples of alcohol-dependent patients and performed non-targeted metabolomic profiling analysis to identify alterations of key metabolites in the plasma of BA-ADPs. Machine learning algorithms and bioinformatic analysis were also used to identify predictive biomarkers and investigate their possible roles in brain atrophy related to alcohol dependence.

**Results:**

A total of 26 plasma metabolites were significantly altered in the BA-ADPs group when compared with a group featuring alcohol-dependent patients without brain atrophy (NBA-ADPs). Nine of these differential metabolites were further identified as potential biomarkers for BA-ADPs. Receiver operating characteristic curves demonstrated that these potential biomarkers exhibited good sensitivity and specificity for distinguishing BA-ADPs from NBA-ADPs. Moreover, metabolic pathway analysis suggested that glycerophospholipid metabolism may be highly involved in the pathogenesis of alcohol-induced brain atrophy.

**Conclusion:**

This plasma metabolomic study provides a valuable resource for enhancing our understanding of alcohol-induced brain atrophy and offers potential targets for therapeutic intervention.

## Introduction

Excessive and chronic alcohol consumption, caused by addictive behaviors in alcoholic patients, is closely related to the reduced viability of neuronal cells (neurons and glial cells) and axonal degradation, thus resulting in brain atrophy (Sutherland et al., 2014; Angebrandt et al., 2022). It has been reported that the degree of brain atrophy correlates with the rate and amount of alcohol consumed over a lifetime (de la Monte and Kril, 2014). Moreover, the latest research has detected negative relationships between alcohol consumption and gray and white matter volumes across the brain (Daviet et al., 2022). Abnormal patterns of macroscopic and microstructural changes in the brain, especially brain atrophy, are closely related to the cognitive dysfunction of alcoholics in the clinical setting (Zahr et al., 2011). Cognitive impairment, including deficits in memory, executive abilities, visuospatial processing, speed of processing and, to a lesser extent, attention and general intelligence, may dramatically influence a patient’s social function and quality-of-life (Godin et al., 2019). In view of the high prevalence of alcohol-related brain atrophy (ARBA) and its associated cognitive dysfunction, a comprehensive analysis of the mechanisms underlying ARBA and the identification of potential biomarkers for this disease are urgently needed.

Metabolomics, a systematic method for the qualitative and quantitative analysis of all low-molecular-weight metabolites, is suitable for identifying metabolic indicators and can provide a basis for individualized diagnosis and treatment (Ribbenstedt et al., 2018). Moreover, the discovery of new markers can provide new ideas for the diagnosis and treatment of difficult diseases and can provide a useful guide for clinical diagnosis and treatment (Vuckovic, 2018). Metabolomic research based on untargeted/targeted mass spectrometry (MS) and proton nuclear magnetic resonance (1H-NMR) spectroscopy approaches may represent a valuable research tool to identify the underlying pathogenesis of alcohol-related disorders. Mittal and Dabur previously studied the influence of an aqueous extract of *Tinospora cordifolia* on the urinary metabolic signature of chronic alcohol using liquid chromatography–tandem mass spectrometry (LC–MS/MS; Mittal and Dabur, 2015). In another study, Zhu et al. identified discriminatory metabolic profiles between healthy and alcohol dependent individuals by using metabolomics technology (Zhu et al., 2021). To our knowledge, no previous study has identified alterations in the metabolic and protein profiles of plasma samples taken from alcohol-dependent patients with brain atrophy.

Machine learning, as a field of artificial intelligence (AI), provides intelligent data processing while facilitating reasoning and the initial settings to determine functional relationships (Deo, 2015). Due to the diversification of algorithms, machine learning is gradually emerging in the field of multi-omics, including artificial neural networks (ANNs), and random forest (RF) algorithms (Liebal et al., 2020). The main applications of machine learning in disease-related multi-omics data analysis include (1) the stratification of patients to discover various subtypes of human diseases and to discover different treatment/prognostic outcomes, and (2) the investigation of various diseases by identifying biomarkers of omics features under various state (Nicora et al., 2020). Traditional methods for processing metabolomics data tend to only focus on bridging sample differences within groups. However, in applied pharmaceutical research (such as candidate target discovery and drug sensitivity), we also need to consider data perturbation and sensitivity to sample size (Schrimpe-Rutledge et al., 2016). Based on their specific characteristics, a combination of traditional single evaluation and machine learning algorithms could provide an efficient means of evaluating the performance of metabolomics data processing from multiple perspectives (Mirza et al., 2019; Picard et al., 2021). Specifically, this strategy can achieve effective data processing from five relatively independent directions: reducing within-group sample differences, differential metabolic analysis, the stability of marker identification, classification accuracy, and the consistency of biological gold standards.

In this study, we used a LC–MS/MS-based metabonomics approach to provide a robust technical platform to investigate the profiles of plasma metabolites in ARBA patients and identify characteristic metabolites that can be used to discriminate ARBA from non-ARBA. MetaboAnalyst version 5.0 was then used to identify metabolites and metabolic pathways showing significant enrichment in ARBA. Then, machine learning algorithms were used to identify the most important distinctive metabolites that might be associated with patients with ARBA. The findings of the present study may help to identify the molecular mechanisms that underlie ARBA.

## Materials and methods

### Study design and participants

This study was approved by the Ethics Committee of the Hunan Brain Hospital (Reference: 2016121). Signed and informed consent was obtained from each patient.

A total of 126 patients with alcohol addiction were enrolled from Hunan Brain Hospital between March 2019 and January 2020. Brain MRI were performed in all patients to evaluate the extent of brain atrophy.

The inclusion and exclusion criteria were described in our previous publication (Liu et al., 2020). Briefly, the inclusion criteria were as follows: (1) age 18–60 years, Han Chinese; (2) no contraindications for MRI. The exclusion criteria were as follows: (1) patients had any general medical conditions or neurological disorders, including infectious, hepatic, or endocrine disease; (2) patients with a history of severe head injury with skull fracture or loss of consciousness of more than 10 min; (3) patients had any current or previous psychiatric disorder; (4) patients had a family history of psychiatric disorder. The diagnosis of alcohol dependence was made according to the Structured Clinical Interview (SCID) based on the Diagnostic and Statistical Manual of Mental Disorders DSM-IV criteria (Battle, 2013). Alcohol-dependent patients were divided into two subgroups based on whether they have brain atrophy (the experimental group) or not (the control group).

### Evaluation of brain atrophy

**Figure 1:**
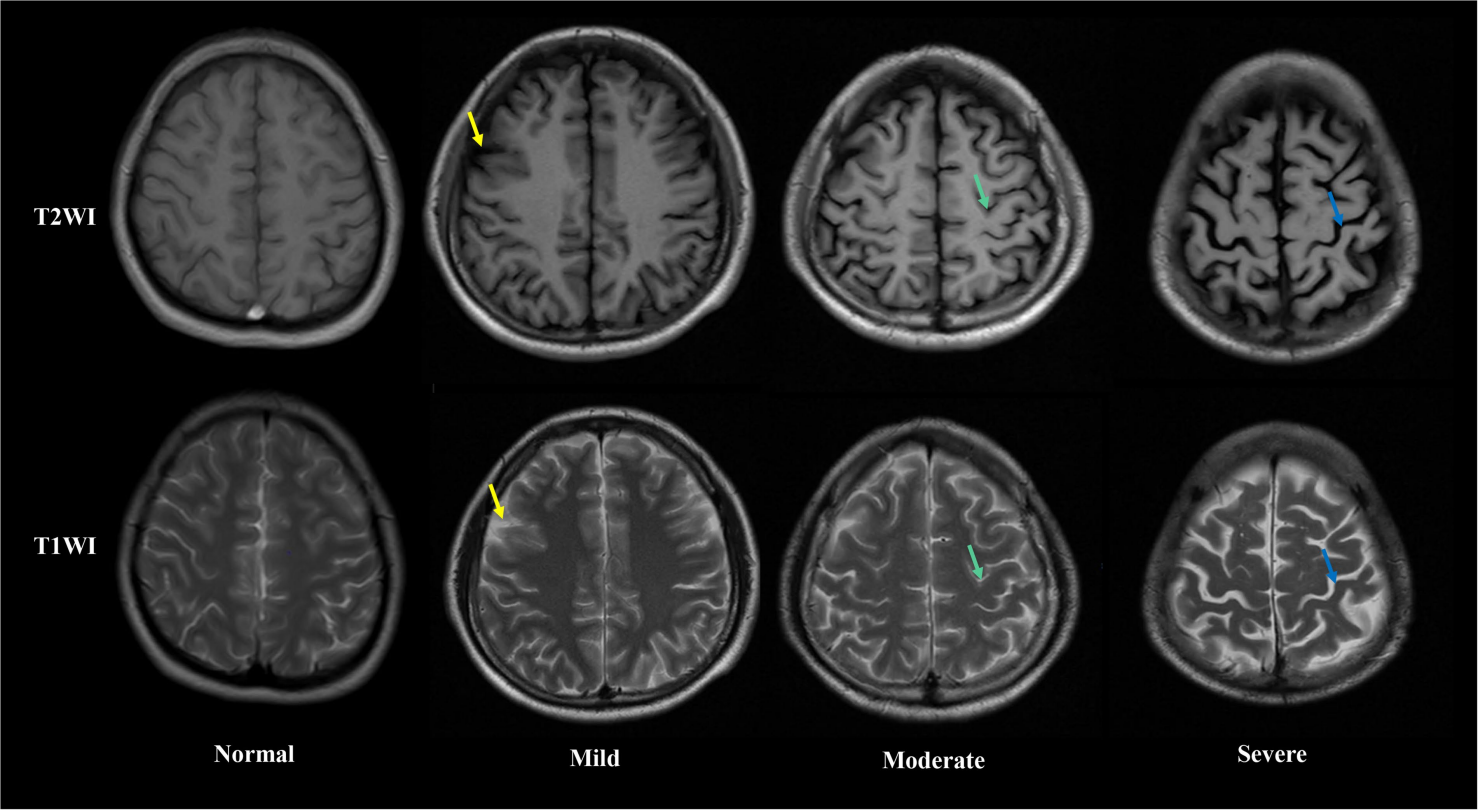
Axial T1 weight (up) and T2 weight (down) magnetic resonance images showing different extent of brain atrophy in Alcohol-dependent patients. From left to right, columns represent absent, low, moderate and sever brain atrophy. In low brain atrophy, sulcal opening peripherally (yellow arrows) is observed. In moderate brain atrophy, widening along the length of the sulcus (green arrows) are observed. In severe brain atrophy, gyral thining (red arrows) is observed.

Brain MRI were performed in all patients after blood samples collection. The extent of brain atrophy was evaluated by at least two independent neuroradiologists using the global cortical atrophy scale (Pasquier et al., 1996). Both cortical regions (frontal, temporal, parietal and occipital) and subcortical regions (peri-insular, basal, and vault) were assessed. The severity of atrophy (low, moderate, or severe) was detected by the widening of sulci and narrow of gyri, as well as the reduction in amplitude of the respective regions. Figure 1 shows examples of different severity of brain atrophy.

### Sample collection and preparation

Blood samples were taken from all inpatients after hospital admission but prior to starting treatment. Blood samples were taken from the experimental and control patients between 6:00 and 6:30 am and placed into plasma collecting tubes. Samples were then centrifuged at 3,000 rpm for 10 min at 4°C and the plasma was aliquoted into 1.5 ml microcentrifuge tubes and immediately stored at −80°C.

For LC–MS, we removed 100 μl of each plasma sample and added 0.4 ml of pre-cooled 0.2% methanol-acetonitrile mixture (1:1, v/v); this was followed by vortex-mixing and ultrasonic extraction on iced water. Next, the solution was centrifuged at 13,000 × *g* for 15 min at 4°C; 400 μl of the supernatant was taken and dried with nitrogen. Finally, 100 μl of acetonitrile water (1:1, v/v) was added and the re-dissolved solution was injected into a sample bottle for detection. Quality control samples (a mixture of equal quantities of all sample extractions) were injected after every 10 analytical samples to monitor the stability of the LC–MS system.

### LC–MS data acquisition and processing

Data acquisition was performed by UPLC-Q-TOF-MS/MS with the following parameter settings: an ethylene bridged hybrid C18 column (2.1 mm × 100 mm id, 1.7 μm; Waters), mobile phase A (water with 0.1% formic acid), and mobile phase B (acetonitrile with 0.1% formic acid). The gradient of the mobile phase was consistent with our previous metabolomic studies (Zhang et al., 2020). The MS signal was acquired in positive-ion and negative-ion modes.

Raw files for the acquired LC–MS/MS data were imported into the metabolomics-processing software Progenesis QI (Waters) to obtain matched and aligned peak data. Subsequently, peak data containing retention time (RT), molecular formula, along with accurate relative molecular mass and peak area information, were imported into Microsoft Excel so that we could normalize the peak area for further analysis.

### Analyses of metabolomics data and pathways

Following normalization of the peak area, data were subjected to principal component analysis (PCA), partial least squares discriminant analysis (PLS-DA), and orthogonal partial least squares discriminant analysis (OPLS-DA). The variables showing the most significant differences were identified by selecting those with a VIP > 1 from an S-plot and *p* < 0.05 from an independent samples t-test. Based on the Human Metabolome Database (HMDB) and secondary fragment ions, we were then able to identify differential variables. Then, we performed metabolic pathway analysis for differentially expressed metabolites with MetaboAnalyst version 5.0 to gain insight into the pathogenesis of ARBA, as described previously (Zhang et al., 2020).

### Machine learning methods for biomarker screening

Three machine learning methods (Extreme Gradient Boosting (XGBoost), random forest (RF), and AdaBoost Classifier) were used to identify potential biomarkers from differential metabolites or proteins. The five most important metabolites were identified by XGBoost, RF and AdaBoost, and then combined for subsequent analysis. Machine learning was then performed using the Extreme Smart Analysis Platform.^1^

The sensitivity and specificity of the combined biomarkers were further analyzed using logistic regression analysis and receiver operating characteristic (ROC) curves. In ROC analysis, the area enclosed by the curve and the *x*-axis (*x* = 1 line) was defined as the area under the curve (AUC). Logistic regression analysis was performed using OmicStudio.^2^

## Results

### Baseline characteristics of the study population

A total of 226 participants were recruited for the present study: 62 were assigned to the NBA-ADP group, 64 were assigned to the BA-ADP group, and 100 were assigned to the Healthy Control (HC) group. Clinical and demographic characteristics were summarized in Table 1. The sex, age, and duration of alcohol dependence between NBA-ADP and BA-ADP group were equivalent. There was no statistical difference in Repeatable Battery for the Assessment of Neuropsychological Status (RBANS) scores between two groups. However, the RBANS scores (including attention, delay memory, immediate memory, language, visuospatial) in BA-ADP group were all slightly higher than these in NBA-ADP group.

**Table 1:** Demographic and baseline patient characteristics.

Characteristics	NBA-ADPs	BA-ADPs
	*n* = 62	*n* = 64
Gender		
Male (*n*)	62	64
Female (*n*)	0	0
Age (years)	40.71 ± 9.01	38.63 ± 9.21
Duration of alcohol dependence (years)	11.94 ± 6.80	13.11 ± 8.63
Duration of alcohol dependence (g) *	147.02 ± 67.81	153.41 ± 71.06
RBANS total score	77.87 ± 12.33	77.58 ± 8.08
Attention	84.79 ± 13.99	83.48 ± 9.86
Delay memory	78.47 ± 19.52	77.04 ± 16.57
Immediate memory	73.19 ± 11.90	73.03 ± 15.30
Language	78.56 ± 11.83	76.90 ± 13.56
Visuospatial	76.14 ± 14.65	75.63 ± 13.47

Abbreviations: NBA-ADPs, Alcohol-dependent patients (ADP) without brain atrophy; BA-ADPs, Alcohol-dependent patients (ADP) with brain atrophy; HC, Heathy controls. RBANS, Repeatable Battery for the Assessment of Neuropsychological Status. Data are shown as mean ± SD.

* *p* < 0.05.

### Global metabolomic profiling

**Figure 2:**
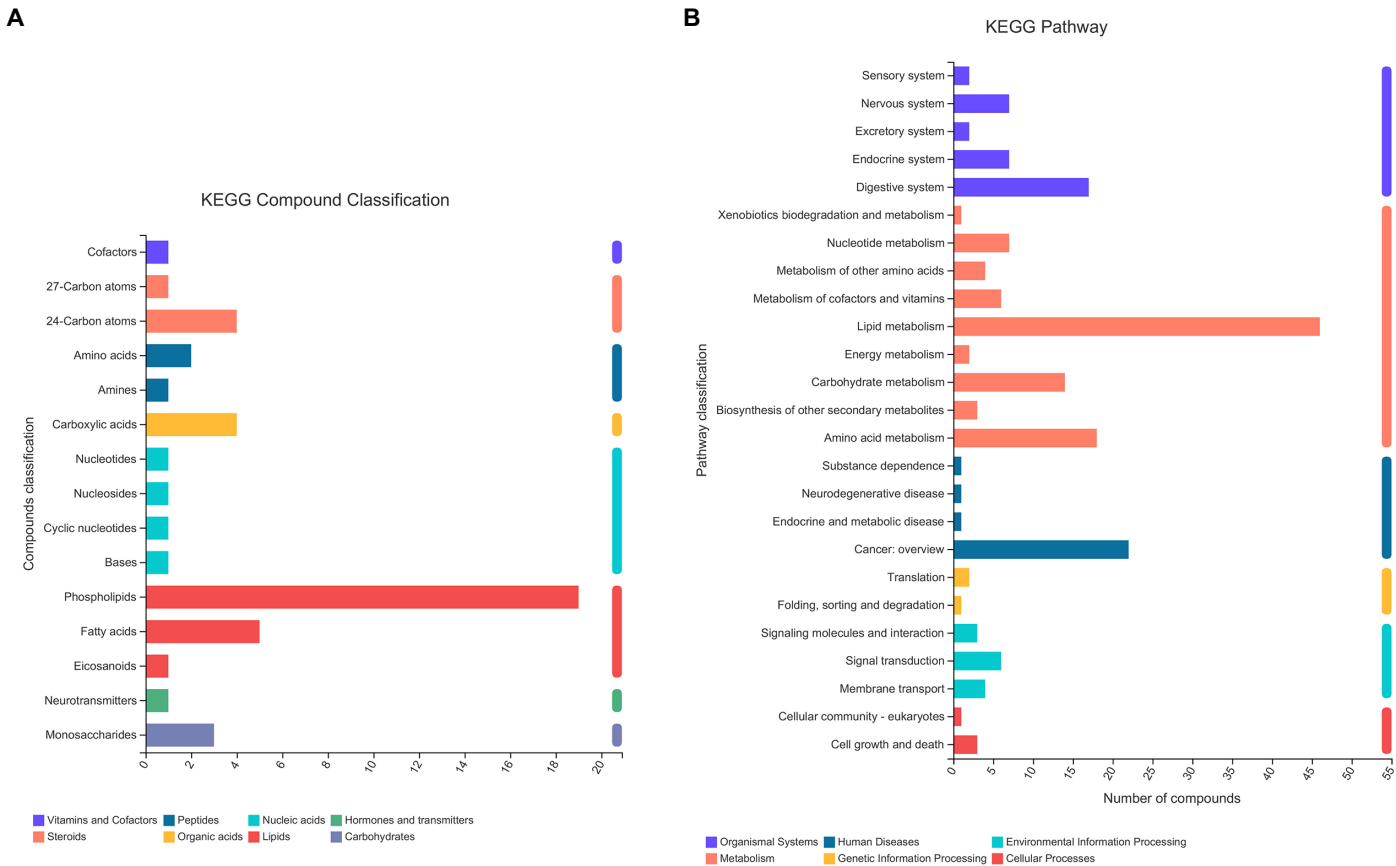
The KEGG classification of metabolites identified in all plasma samples. The classification criteria were: **(A)** KEGG Compound Classification; **(B)** KEGG Pathway.

A total of 13,601 peaks were obtained, including 6,189 positive-mode features and 8,432 negative-mode features. A total of 178 positive-mode and 253 negative-mode metabolites were annotated and mapped to public databases. Corresponding to the two modes, 52 and 84 metabolites were, respectively, annotated and mapped to the Kyoto Encyclopedia of Genes and Genomes (KEGG) database. These metabolites belonged to 15 different KEGG compound classifications. Figure 2A shows that these metabolites were mainly classified as phospholipids. According to the KEGG database, these metabolites belonged to 25 different KEGG pathways (Figure 2B). Of these, the lipid metabolism pathway was the pathway that contained the most metabolites.

**Figure 3:**
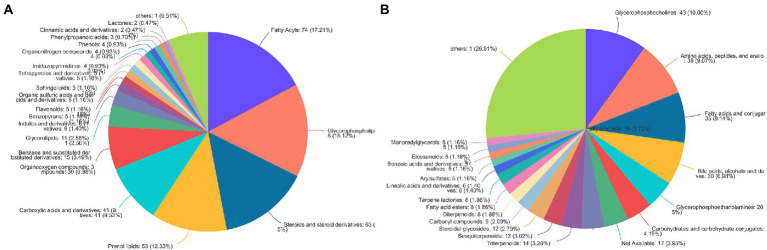
The HMDB classification of metabolites identified in all plasma samples. Pie chart illustrating the abundance ratio of different classes of plasma metabolites detected with untargeted metabolic profiling. The classification level was: **(A)** Superclass; **(B)** Subclass.

According to the HMDB version 4.0 database, 431 selected metabolites belonged to 9 predominant super-classes (Figure 3A) and 21 subclasses (Figure 3B). The former included lipids and lipid-like molecules (63.03%), organic acids and derivatives (11.61%), organoheterocyclic compounds (7.21%), and organic oxygen compounds (6.98%). The latter includes glycerophosphocholines (10.00%), amino acids, peptides, and analo (9.07%), fatty acids and conjugat (8.14%), bile acids, alcohols and deves (6.98%), and glycerophosphoethanolamines (5%).

### Identification of dysregulated metabolites

**Figure 4:**
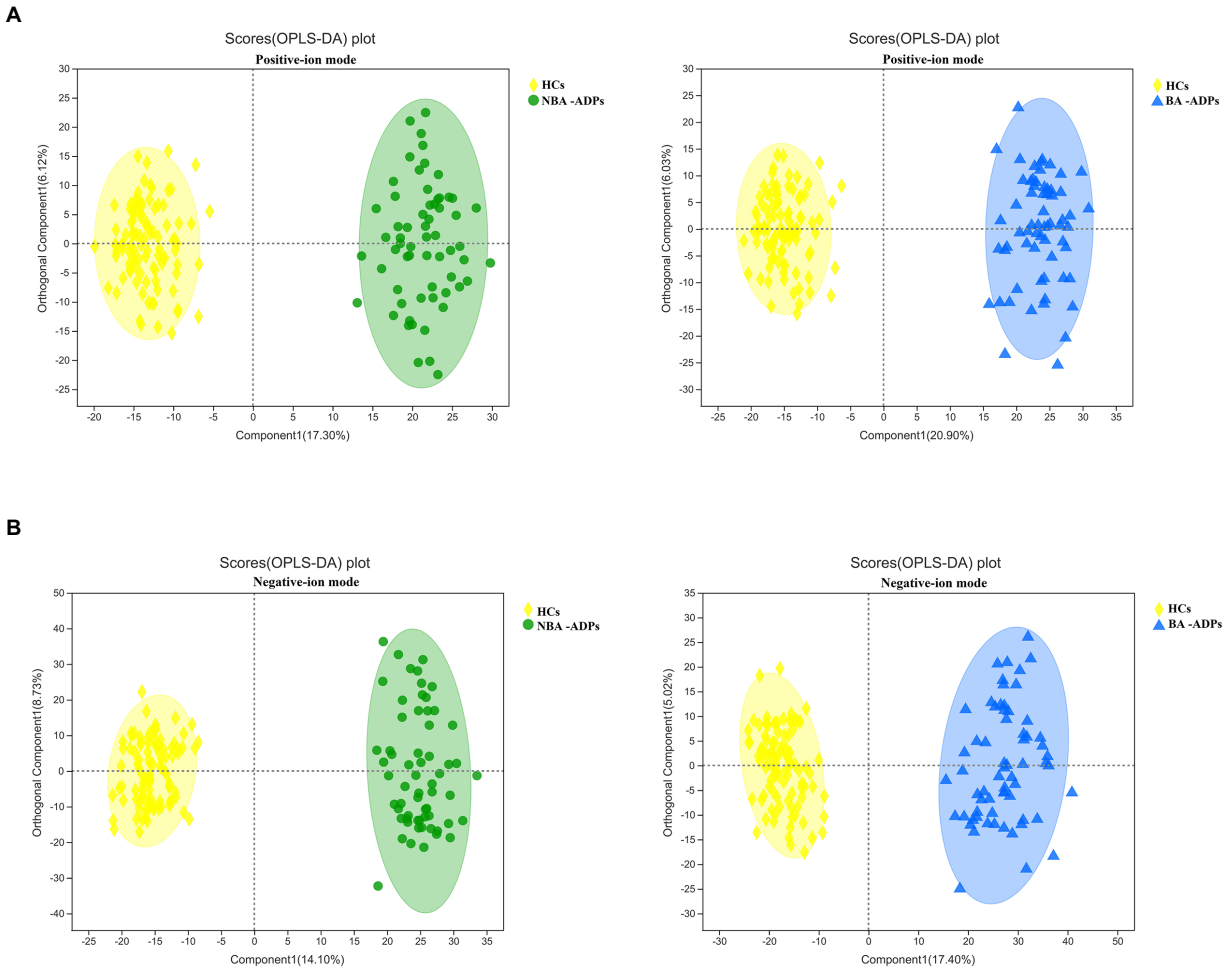
OPLS-DA score plots of alcohol-dependent patients (ADP) versus healthy controls. **(A)** OPLS-DA score plots of NBA-ADP vs. HC; **(B)** OPLS-DA score plots of BA-ADP vs. HC.

A total of 172 significant differential metabolites (FDR < 0.05) among the three groups were detected by analysis of variance (ANOVA). To further identify group differences in the metabolic profiles between groups, we performed OPLS-DA score plots; these identified notable separations between both the BA-ADP and HC group and between the NBA-ADP and HC group (Figure 4).

**Figure 5:**
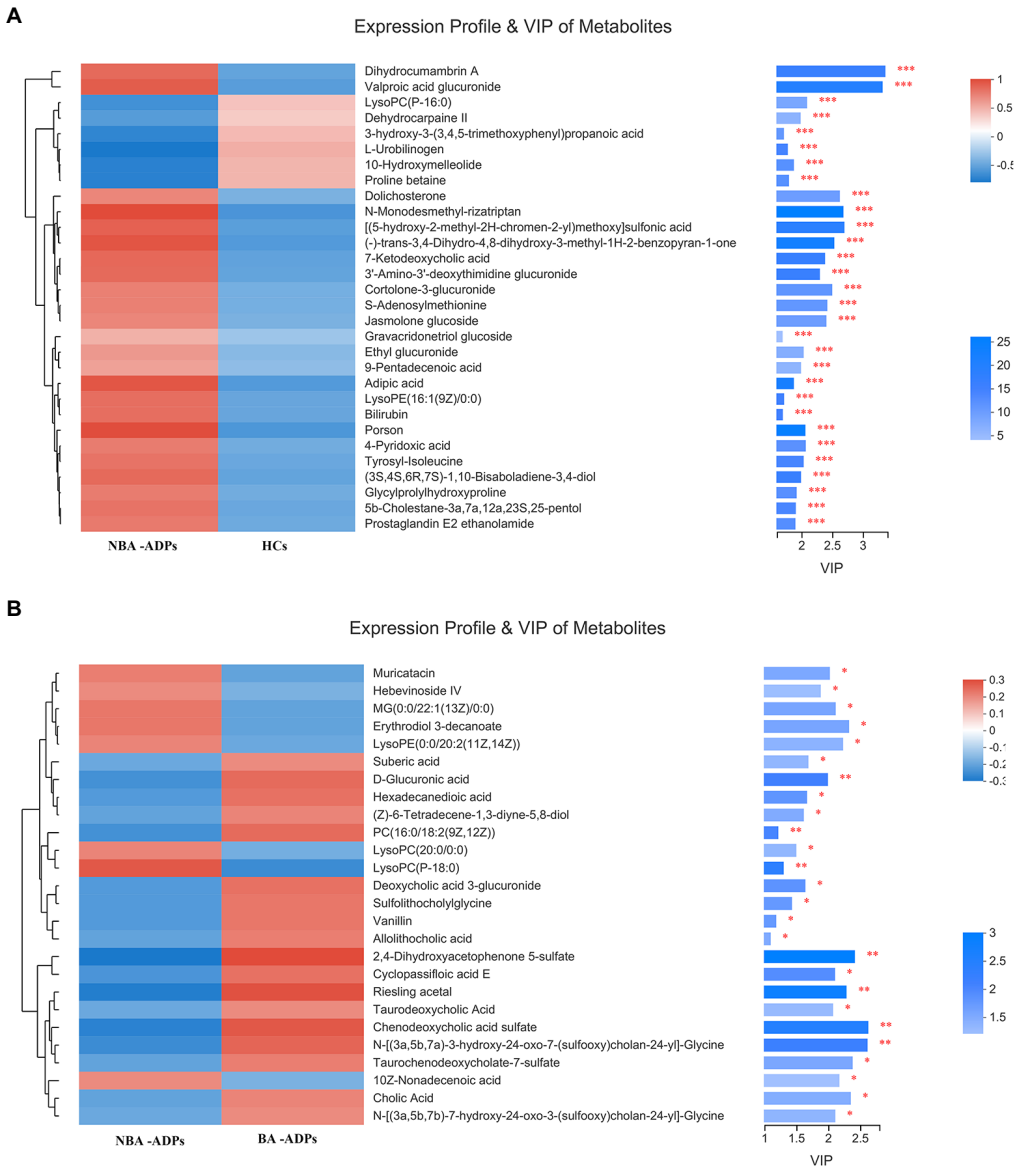
Differential plasma metabolic profiles of alcohol-dependent patients vs. healthy controls. The hierarchical clustering and heat map in the left panel shows the top 30 metabolites that were significantly differentially abundant between NBA-ADP and HC **(A)**, and 26 metabolites that were significantly differentially abundant between BA-ADP and NBA-ADP **(B)**. The histogram in the right panel represents variable importance in projection (VIP) scores derived from the OPLS-DA model for each metabolite. ∗∗∗ indicates P < 0.001.

Differentially expressed metabolites were identified using multivariate and univariate statistical significance criteria (VIP > 1 and FDR < 0.05). In total, 139 metabolites were identified to be significantly different between the NBA-ADP and HC group and 26 metabolites between the BA-ADP and NBA-ADP group (Figure 5; Table 2).

**Table 2:** Differentially expressed endogenous metabolites detected by UHPLC-QTOF/MS.

Metabolite	Library ID	M/Z	Formula	RT	log2FC	-log10(p_value)
Positive-ion mode						
Taurodeoxycholic Acid	HMDB0000896	464.282	C26H45NO6S	5.113	0.074	1.394
LysoPC(P-18:0)	HMDB0013122	508.375	C26H54NO6P	8.648	0.017	2.404
Cholic Acid	HMDB0000619	373.273	C24H40O5	5.377	0.06	1.555
Hebevinoside IV	HMDB0036060	587.427	C36H60O7	9.547	−0.016	1.329
PC(16:0/18:2(9Z,12Z))	HMDB0007973	758.568	C42H80NO8P	10.753	0.007	2.103
Allolithocholic acid	HMDB0000381	394.33	C24H40O3	9.127	0.035	1.599
Muricatacin	HMDB0038685	591.459	C17H32O3	9.57	−0.016	1.626
LysoPC(20:0/0:0)	HMDB0010390	574.383	C28H58NO7P	9.48	−0.021	1.432
Erythrodiol 3-decanoate	HMDB0034510	635.482	C40H68O3	9.326	−0.03	1.672
Negative-ion mode						
Sulfolithocholylglycine	HMDB0002639	512.269	C26H43NO7S	6.36	0.072	1.821
N-[(3a,5b,7a)-3-hydroxy-24-oxo-7-(sulfooxy)cholan-24-yl]-Glycine	HMDB0002496	528.264	C26H43NO8S	4.687	0.066	2.229
N-[(3a,5b,7b)-7-hydroxy-24-oxo-3-(sulfooxy)cholan-24-yl]-Glycine	HMDB0002409	528.264	C26H43NO8S	5.624	0.044	1.456
Taurochenodeoxycholate-7-sulfate	HMDB0002498	288.62	C26H45NO9S2	4.118	0.078	1.636
Chenodeoxycholic acid sulfate	HMDB0002522	471.242	C24H40O7S	6.13	0.104	2.529
Hexadecanedioic acid	HMDB0000672	285.207	C16H30O4	7.344	0.041	1.884
Riesling acetal	HMDB0037562	271.155	C13H22O3	4.938	0.098	2.829
Suberic acid	HMDB0000893	173.081	C8H14O4	2.983	0.029	1.426
D-Glucuronic acid	HMDB0000127	193.035	C6H10O7	0.614	0.034	2.152
Cyclopassifloic acid E	HMDB0036298	597.365	C31H52O8	6.291	0.124	1.981
10Z-Nonadecenoic acid	HMDB0013622	341.27	C19H36O2	7.023	−0.123	1.312
MG(0:0/22:1(13Z)/0:0)	HMDB0011552	457.353	C25H48O4	9.031	−0.041	1.669
LysoPE(0:0/20:2(11Z,14Z))	HMDB0011483	550.314	C25H48NO7P	7.504	−0.054	1.487
Deoxycholic acid 3-glucuronide	HMDB0002596	567.318	C30H48O10	5.601	0.057	1.904
Vanillin	HMDB0012308	151.039	C8H8O3	2.576	0.064	1.811
2,4-Dihydroxyacetophenone 5-sulfate	HMDB0041646	230.996	C8H8O6S	2.576	0.157	2.999
(Z)-6-Tetradecene-1,3-diyne-5,8-diol	HMDB0038996	255.114	C14H20O2	1.707	0.052	1.586

Abbreviations: FC: fold change, as determined by average relative quantitation obtained from group BA-ADPs/NBA-ADPs; RT (min): retention time.

### Functional analysis of differentially expressed plasma metabolites from alcohol-dependent patients

To gain a further understanding of the metabolic disturbances between the NBA-ADP and HC group and between the BA-ADP and NBA-ADP group, we performed KEGG pathway enrichment analysis; we also used MetaboAnalyst version 5.0 to perform a functional analysis of plasma metabolites.

**Figure 6:**
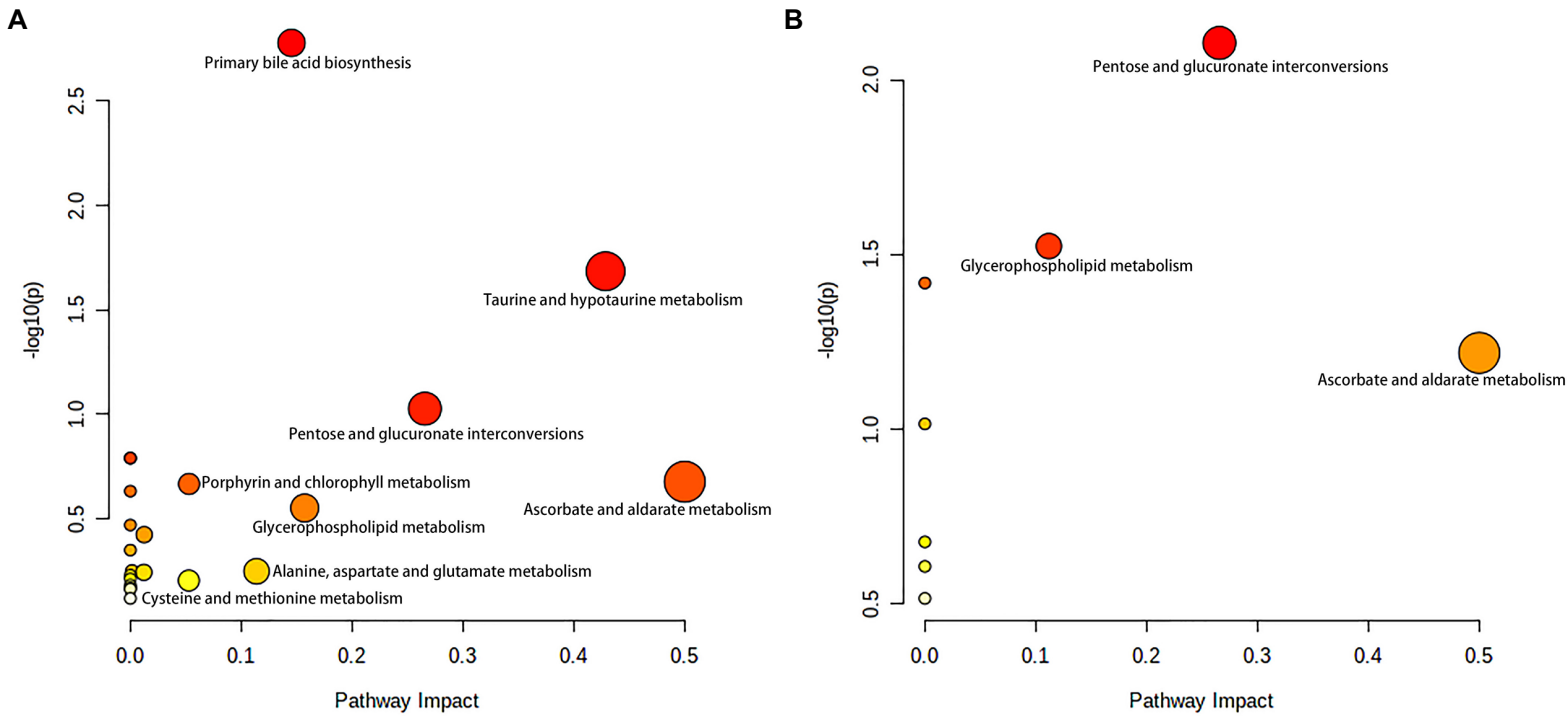
Metabolite pathway changes identified using MetaboAnalyst 5.0. Pathway analysis of the differential plasma metabolites between NBA-ADP vs. HC **(A)**, and BA-ADP vs. NBA-ADP **(B)**. The y axis shows the *p*-values and the x axis, representing pathway impact values; node color is based on its *p*-value and node size reflects the pathway impact values.

As shown in Figure 6A, primary bile acid biosynthesis (map00120), taurine and hypotaurine metabolism (map00430) were the most important metabolic pathways that showed alterations in the alcohol-dependent patients (both the NBA-ADP group and the BA-ADP group) when compared with the HC group. In contrast, pentose and glucuronate interconversions (map00040) and glycerophospholipid metabolism (map00564) were detected in the BA-ADP group when compared with the NBA-ADP group (Figure 6B).

### Screening of potential metabolic biomarkers for alcohol-dependent patients with brain atrophy

**Figure 7:**
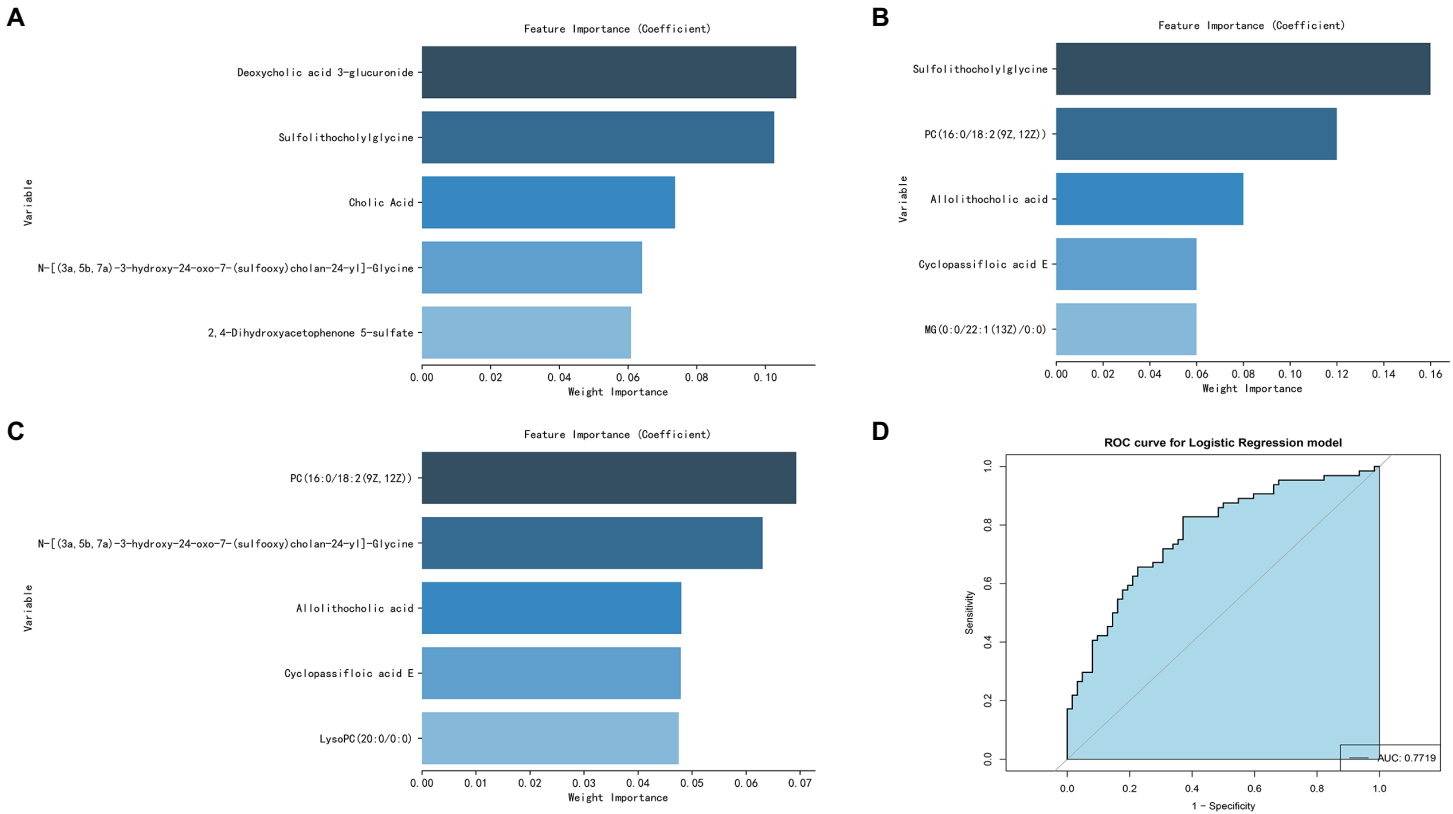
Screening of potential plasma metabolite biomarkers of alcohol-dependent patients with brain atrophy. The nine most important metabolites selected by Naive Bayes **(A)**, AdaBoost **(B)**, and Random Forest **(C)**. The AUC value of the ROC curve of potential plasma metabolite biomarkers for distinguishing BA-ADP patients from NBA-ADP **(D)**.

The five most important metabolites selected by AdBooST were Sulfolithocholylglycine PC (16:0/18:2(9Z,12Z)), Allolithocholic acid, MG(0:0/22:1(13Z)/0:0), and Cyclopassifloic acid E (Figure 7A). The five most important metabolites selected by Random forest were PC (16:0/18:2(9Z,12Z)), N-[(3a,5b,7a)-3-hydroxy-24-oxo-7-(sulfooxy)cholan-24-yl]-Glycine, Cyclopassifloic acid E, 2,4-Dihydroxyacetophenone 5-sulfate, and Allolithocholic acid (Figure 7B). The five most important metabolites selected by Naive Bayes were Deoxycholic acid 3-glucuronide, Sulfolithocholylglycine, Cholic Acid, N-[(3a,5b,7a)-3-hydroxy-24-oxo-7-(sulfooxy)cholan-24-yl]-Glycine, and 2,4-Dihydroxyacetophenone 5-sulfate (Figure 7C). Nine metabolites (Cholic Acid, PC (16:0/18:2(9Z,12Z)), allolithocholic acid, sulfolithocholylglycine, N-[(3a,5b,7a)-3-hydroxy-24-oxo-7-(sulfooxy)cholan-24-yl]-Glycine, cyclopassifloic acid E, MG(0:0/22:1(13Z)/0:0), deoxycholic acid 3-glucuronide, 2,4-Dihydroxyacetophenone 5-sulfate) were identified as potential metabolic biomarkers for alcohol-dependent patients with brain atrophy. As shown in Figure 7D, the AUC of the ROC curve reached was 0.7719 for distinguishing between BA-ADP and NBA-ADP patients.

## Discussion

It is well-known that alcohol can causes serious health problems, long-term abuse, and irreversible alterations in the structure and function of the brain (Rehm et al., 2017; Kranzler and Soyka, 2018). However, to the best of our knowledge, this is the first study that sought to identify potential plasma biomarkers of alcohol-dependent patients who have brain atrophy by applying non-targeted metabolomics and machine learning.

In this study, a total of 26 metabolites were found to show significant changes between the BA-ADP and NBA-ADP group. Each of the identified metabolites were searched against synonyms in HMDB. These metabolites covered a range of chemical classes; lipids and lipid-like molecules were ranked highest and featured 22 of the metabolites. The obtained metabolites were classified based on the KEGG compound annotation database.^3^ Phospholipids and phospholipid metabolism were ranked first in the secondary classification category of KEGG compounds. As the main substance of the brain, lipids not only act as the building blocks of all membrane structures but also act as the repository for chemical energy and play a significant role in cellular signaling pathways (Holthuis and Menon, 2014; Van Deijk et al., 2017). Characteristic alterations in lipid—including structure, composition, or distribution—are thought account for alterations in neuronal function, synaptic signaling, and neurotransmitter transmission. A previous lipidomics study investigating the links between chronic alcohol infusion and whole brain lipid profile, demonstrated that specific lipid categories, mainly PS, PC and PE, appeared to be related to neuro-pathology (Wang et al., 2019). To further identify the potential metabolic biomarkers of BA-ADP, machine learning was used and nine metabolites ((Cholic Acid, PC (16:0/18:2(9Z,12Z)), Allolithocholic acid, Sulfolithocholylglycine, N-[(3a,5b,7a)-3-hydroxy-24-oxo-7-(sulfooxy) cholan-24-yl]-Glycine, Cyclopassifloic acid E, MG(0:0/22:1(13Z)/0:0), Deoxycholic acid 3-glucuronide, 2,4-Dihydroxyacetophenone 5-sulfate) were identified. The AUC of these metabolite biomarkers was 0.7719, thus indicating an acceptable correlation using metabolite biomarkers and an outstanding correlation using protein biomarkers.

Of the nine differential metabolites identified in this study, there were several different species of bile acids, including cholic acid, allolithocholic acid, and deoxycholic acid 3-glucuronide. Bile acids readily cross the blood–brain barrier and their receptors are expressed in central tissues, suggesting that they may have important functional roles (Romanazzi et al., 2021). The bile acid signaling pathway plays an extremely important role in diseases and is a target for drug intervention. Drugs related to bile acids include chenodeoxycholic acid and its derivatives, ursodeoxycholic acid and its derivatives, bile acid sequestrants (such as cholestyramine), and apical sodium-dependent bile acid transporter (ASBT) inhibitors. In the nervous system, ASBT-mediated bile acid reabsorption significantly increases the level of bile acid in the serum and brain tissue, reduces the acidity of the intestinal cavity, increases the pH of the intestine, and promotes the conversion of intestinal ammonium into ammonia, thus resulting in abnormally increased levels of neurotoxic ammonia and cytotoxic bile acid in the blood and brain. Previous studies have confirmed that changes in bile acid occur in patients who progress from cognitive impairment to Alzheimer’s disease, and the relationship between this change and cognitive decline is well documented. In addition, our study identified two metabolites related to bile acids. The expression of these two-acyl glycine and bile acid-glycine conjugates varied significantly between the BA-ADP and NBA-ADP group. Therefore, we assumed that bile acids may strongly associated with alcohol-related brain atrophy. And additional targeted absolute quantitative analyze of the bile acid spectrum in future studies may be helpful.

Interestingly, another class of potential metabolic biomarkers here we identified are lipid metabolites. It is widely documented that the homeostasis of lipid metabolism plays a significant role in the central nervous system. Many lipidomic-based studies have reported the relationship between the dysregulation of specific lipids and pathological conditions, including diabetes, Alzheimer’s disease, hypertension, and cancer. It has been reported that long-term alcohol exposure significantly modifies the serum lipid profile, especially the metabolic pathways involving glycerophospholipid, sphingolipid and glycerolipids. Alcohol exposure can dramatically influence the lipidome of both the prefrontal cortex and striatum, thus leading to alcohol-related neurotoxicity and neuroplasticity. In the present study, by applying metabolomics analysis, we found that some glycerophospholipid (GP) metabolites, such as PC(P-16:0/18:2(9Z,12Z)), were significantly altered in alcohol-dependent patients with brain atrophy. GPs are the main components of the membrane structure. Different cell types, organelles, and inner/outer membranes in mammalian mitochondria, are known to have distinct glycerophospholipid compositions; these differences relate to the specific biological functions of these structures (Klaming et al., 2019). As GPs provide neural membranes with stability, fluidity and permeability, they are necessary for the normal biological function of integral membrane proteins, receptors, and ion-channels. Our present results suggest that chronic alcohol exposure may lead to brain atrophy and affect brain functionality by altering the composition of GPs. Alterations of the GP composition in neural membranes could therefore be related to neurological disorders. In addition, we found that some glycerolipids, such as MG(0:0/22:1(13Z)/0:0), were significantly altered in alcohol-dependent patients with brain atrophy. Further research now needs to investigate the key metabolic enzymes that mediate alcohol-induced dysfunction of lipidome profiling in the brain.

Based on the identified metabolites, we further identified two significantly altered metabolic pathways (primary bile acid biosynthesis, and taurine and hypotaurine metabolism) that were most closely related to alcohol dependence irrespective of whether brain atrophy was involved or not. As endogenous signaling molecules, bile acids are synthesized in the liver and secreted into the gastrointestinal tract for postprandial nutrient absorption and to control the overgrowth of microbial growth. In addition, gut microbes metabolize bile acids and in doing so, determine the composition of the circulating bile acids, thus regulating host metabolism (Chiang and Ferrell, 2019). Patients with acute alcohol intake exhibit increased serum levels of bile acids and cholestatic liver injury; alcohol intake also increases bile acid pool size and reduces bile acid flow and fecal excretion (Donepudi et al., 2018). Wang et al. found that the abundance of firmicutes and clostridium was notably increased in alcohol-addicted mice, and the levels of secondary bile acids produced by firmicutes had increased (Wang et al., 2018). In conclusion, compared with healthy individuals, there are certain changes in the expression levels of bile acid metabolites (including tauroursodeoxycholic acid, cholic acid and allocholic acid) in alcohol-addicted patients. The analysis showed that regulation of bile acid biosynthesis is likely to contribute to the occurrence and development of alcohol-related diseases.

As for taurine and hypotaurine metabolism, taurine has already been shown to protect mice with alcoholic liver injury by reducing hepatic oxidative stress and interrupting the alcohol-induced renal inflammatory cycle (Tang et al., 2019). It is well known that taurine can also prevent and repair liver damage and balance liver lipid metabolism indicators in a mouse model of alcoholic liver disease. The mechanism involved in this protection may be related to the regulation of related enzymes and transcriptional regulators involved in lipid metabolism (Latchoumycandane et al., 2014). In another study, Xia et al. suggested that the metabolic pathways of ascorbic acid, taurine, and hypotaurine, may play an active role in the protection against Antrodin A secreted by *Antrodia camphorata* and thus protect against alcoholic liver injury (Yi et al., 2021). Our present study also found that the BA-ADP group showed an elevation in taurine, compared with the NBA-ADP group. Thus, those imply that the metabolic pathways of taurine and hypotaurine may be also associated with alcohol-related brain atrophy.

To further identify the potential pathways that may be associated with alcohol-related brain atrophy, the KEGG pathway analysis using difference metabolites between BA-ADP and NBA-ADP was performed. We found that glycerophospholipid metabolism, along with pentose and glucuronate interconversions, were significantly associated with alcohol-related brain atrophy. This finding is in line with previous studies that demonstrated the impact of glycerophospholipid metabolism on neurodegenerative changes (Frisardi et al., 2011). Previous studies have shown that the degradation products of glycerophospholipids have pro-inflammatory effects and that their production is often accompanied by the activation of astrocytes and microglia and the release of inflammatory cytokines; these changes lead to oxidative stress and neuroinflammation (Bonelli et al., 2020). Changes in glycerophospholipid metabolism have also been shown to lead to changes in cell membrane permeability and ion homeostasis, thus leading to oxidative stress and neurodegenerative changes (Fuller and Futerman, 2018). Increased small vascular disease load was linked to changes in glycerophospholipid metabolism, as seen by increased white matter hyperintensity volume, decreased mean diffusivity normalized peak height, increased brain atrophy, and decreased cognition (Harshfield et al., 2022). Previous report has found that pentose and glucuronate interconversions is associated with the cognitive impairment in Alzheimer disease (He et al., 2020). In the present study, we report, for the first time, that pentose and glucuronate interconversions are also associated with alcohol-related brain atrophy. However, the precise role of these two regulatory metabolic pathways in the pathophysiological mechanism of alcohol dependence-related brain atrophy requires further investigation.

## Limitations

This study has several limitations that need to be considered. First, the metabolomic analysis performed in the present study did not provide absolute quantification. If this model is to be applied clinically, more rigorous quantification and extensive validation of metabolites would be needed. Targeted metabolomics could be used to validate these specific plasma metabolomic biomarkers. Second, the sample size of this study was rather small, particularly in proteomic analysis; thus, additional patients are required for future analysis. Third, only XGBoost, RF, and AdaBoost Classifier were used to screen for potential biomarkers. Other machine learning methods, such as Support Vector Machine and Boruta could be used in future analyses. Fourth, due to the complex genetic and microenvironmental backgrounds of our patients, other biofluids, such as urine, serum, and cerebrospinal fluid, could also be used to identify additional novel biomarkers. This will provide a more to comprehensive understanding of the pathogenesis of brain atrophy in alcohol-dependent patients. Last, we failed to reveal any differences in cognitive tests between control patients and those identified as having brain atrophy in this study, more detailed cognitive tests may be help in future research.

## Conclusion

This was the first attempt to conduct a metabolomic analysis of plasma samples from healthy control groups and alcohol-dependent patients. Our data showed that patients with alcohol-dependent brain atrophy had distinct metabolic profiles compared with healthy controls and alcohol-dependent patients who do not have brain atrophy. Furthermore, bioinformatic analysis suggested that alterations in the metabolome may be involved in disease pathogenesis. Although further research is needed, our results offer useful diagnostic and therapeutic clues for the management of alcohol-dependent patients with brain atrophy.

## Data availability statement

The original contributions presented in the study are included in the article/Results, and further inquiries can be directed to the corresponding author.

## Ethics statement

The studies involving human participants were reviewed and approved by The Ethics Committee of the Hunan Brain Hospital (Reference: 2016121). The patients/participants provided their written informed consent to participate in this study.

## Author contributions

SZ and XHZ designed the study. XC, CH, ZL, XW, XL, QL, XZ, YG, HX, and TX managed the data collection. SZ, JH, ZZ, and XHZ undertook the statistical analysis. ZZ wrote the first draft of the manuscript. All authors contributed to the article and approved the submitted version.

## Funding

This work was financially supported by the National Key Research and Development Program of China (No. 2018YFC1314401), Hunan Provincial Natural Science Foundation of China (Nos. 2018JJ3284, 2020JJ4803, 2022JJ2540, 2022JJ2615, 2021JJ70013, 2021JJ70089, and 2022JJ40728), The Scientific Research Project of the Hunan Health Commission (Nos. A202303096949, 20200620, and 202103092373), Special Project on Science and Technology Development and First-class Discipline Construction of Clinical Medicine Discipline of Hunan University of Chinese Medicine in the 13th Five-Year Plan Period (2091007), and Key Clinical Specialty Construction Project of the Hunan Health Commission (Improvement of Diagnosis and Treatment Ability of Severe Psychiatric Diseases in Hunan Province).

## Conflict of interest

The authors declare that the research was conducted in the absence of any commercial or financial relationships that could be construed as a potential conflict of interest.

## Publisher’s note

All claims expressed in this article are solely those of the authors and do not necessarily represent those of their affiliated organizations, or those of the publisher, the editors and the reviewers. Any product that may be evaluated in this article, or claim that may be made by its manufacturer, is not guaranteed or endorsed by the publisher.

## Footnotes

^1^ https://www.xsmartanalysis.com/

^2^ https://www.omicstudio.cn/tool/58

^3^ https://www.kegg.jp/kegg/compound/
